# Recent Pain Among Older Women and Men in the United States by Sexual Orientation

**DOI:** 10.1093/geroni/igaa057.1700

**Published:** 2020-12-16

**Authors:** Adena Galinsky, Karen Fredriksen Goldsen, James Dahlhamer, Tina Norris

**Affiliations:** 1 National Center for Health Statistics, Hyattsville, Maryland, United States; 2 University of Washington, Seattle, Washington, United States

## Abstract

Pain is not only a result of other health problems but an independent condition that can negatively impact quality of life. Lesbian, gay, and bisexual older adults report more pain compared to their straight counterparts when pain is measured in the aggregate (e.g. “one or more of the following types of pain”). However, scant national research has examined if specific types of pain vary by sexual orientation among older adults. Using 2015-2018 National Health Interview Survey (NHIS) data, we used logistic regression to separately model four types of pain among women 50+ and men 50+ (lesbian/gay women: n=377, bisexual women: n=142, straight women: n=33,216; gay men: n=508, bisexual men: n=115, straight men: n=25,998) as functions of sexual orientation, controlling for age, race, education, and income. Lesbian women and bisexual men were more likely (AOR=1.46, 95% CI:1.03, 2.08; AOR=2.95, 95% CI:1.08, 3.79, respectively), but bisexual women were less likely (AOR=0.6, 95% CI:0.33, 1.05), than their straight counterparts to have had a migraine or severe headache in the past three months. Bisexual men were also more likely than straight men to report lower back pain in the past three months (AOR=1.66, 95% CI: 1.02, 2.72). Sexual minority women were more likely than straight women to report joint pain in the past 30 days and lower back pain in the past three months. Future research may examine why the prevalence of specific types of pain vary by sexual orientation among older adults.

